# Neurological findings in a cohort of adults with down syndrome

**DOI:** 10.1007/s10072-025-08195-7

**Published:** 2025-05-13

**Authors:** Nicola Grotteschi, Magali Jane Rochat, Virginia Pollarini, Alessandro Ghezzo, Camilla Pellegrini, Giovanna Calandra-Buonaura, Raffaele Lodi, Caterina Tonon, Pietro Cortelli, Maria Giulia Bacalini, Gian Luca Pirazzoli, Luisa Sambati

**Affiliations:** 1 Department of Biomedical and Neuromotor Sciences (DIBINEM), University of Bologna, Bologna, Italy; 2 IRCCS Istituto delle Scienze Neurologiche di Bologna, Bologna, Italy; 3 IRCCS Istituto delle Scienze Neurologiche di Bologna, U.O.C. Clinica Neurologica Rete Metropolitana, Bologna, Italy; 4 Centro Grioni– Fondazione Danielli, Lodi, Italy; 5 Fondazione Madonna della Bomba Scalabrini ETS, Piacenza, Italy

**Keywords:** Down Syndrome, Intellectual disability, Dementia, Down Syndrome Regression Disorder, Psychiatric disorder, Movement disorder

## Abstract

The aim of this study is to describe the results of a comprehensive neurological assessment conducted on a cohort of 70 adults with Down syndrome (DS), aged 21 to 74 years, recruited in Bologna, Italy. Neurocognitive disorder (NcD) was identified in 28.6% of participants and showed a significant association with psychiatric disorders (*p*=0.03). Psychiatric conditions were present in 40% of the cohort, while Down Syndrome Regression Disorder (DSRD) was diagnosed in 7.1%. Transient loss of consciousness affected 28.6% of individuals. Epilepsy, observed in 7.1%, was significantly associated with NcD (*p*=0.02). Neurological examination revealed that stereotypic movements correlated with DSRD (*p*<0.01), tics with obsessive-compulsive disorders (*p*=0.01), and hypokinetic movement disorders with psychotic conditions (*p*=0.03). Additionally, nystagmus and cerebellar signs were significantly associated with elevated serum bilirubin levels (*p*<0.01). These findings underscore the high prevalence and complexity of neurological comorbidities in adults with DS, emphasizing the need for specialized, multidisciplinary care.

**Supplementary Information:**

The online version contains supplementary material available at 10.1007/s10072-025-08195-7.

## Introduction

Down Syndrome (DS) is due to total or partial trisomy of chromosome 21, which results in morphological and functional abnormalities involving multiple organs and systems [1]. Aging with DS is accompanied by a high rate of multimorbidity and polypharmacotherapy, further complicating the clinical presentation of the syndromic condition [2].

Several authors have been interested in the characterization of neurological phenotypes of DS during adulthood [3]. Neurological findings must be assessed within a complex clinical framework, considering neuroanatomical abnormalities, intellectual disability (ID), age-related comorbidities and polypharmacotherapy [3, 4]. Moreover, visual and auditory deficits are common syndromic features which make neurological assessment even more challenging [2].

ID is the core neurological trait of DS, which may present with a wide inter-individual variability; attention is considered one of the most impaired cognitive domains, along with linguistic production and comprehension [5, 6]. The main determinant of ID in DS is likely to be an impaired neurogenesis during foetal life stages, which results in a reduction in the number of neurons [7]. Moreover, this population is at high risk of developing Alzheimer’s disease (AD), possibly due to the overexpression of the *APP* gene, which maps on chromosome 21. Dementia is a major neurological comorbidity with a prevalence that clearly tends to increase with age, starting at 55% between 40 and 50 years, and reaching 100% in patients over 70 years of age [8, 9]. The study of ID and Alzheimer’s Disease in DS is a primary focus of research [5], but other causes of cognitive and functional impairment have been described. Down Syndrome Regression Disorder (DSRD) is a rare condition, with a subacute onset at a young age, characterized by a broad clinical phenotype, dominated by a regression in cognitive abilities and neurological symptoms such as bradykinesia, catatonia and epilepsy [10]. Interest in this condition has grown only in recent years and, due to its rarity, it is still difficult to establish its prevalence. Behavioural disorders are common in DS and it is still challenging to determine whether these symptoms can be classified in specific psychiatric disorders. Due to the lack of specific diagnostic criteria, the clinical study of psychiatric symptoms in DS has been inconsistent, and the prevalence of disorders is still difficult to define [4, 11].

Movement disorders are common in adults with DS although, to the best of our knowledge, few studies provided a systematic description of their occurrence and characteristics [12, 13]. The most frequent findings include orofacial dyskinesias, stereotypic movements and bradykinesia.

Other common neurological disorders are epilepsies, with a prevalence up to 13%, cerebrovascular events, like moyamoya syndrome (3.8%), and sleep disorders like Obstructive Sleep Apnoea Syndrome (OSAS) (13.5%) [2, 4].

Most of the studies on the neurological phenotypes in adults with DS focused on specific conditions or described their contribution to the onset, progression and diagnosis of dementia. The aim of this paper is to further contribute to this field by describing the results of a comprehensive neurological physical examination performed in a cohort of adults with DS recruited in Bologna, Italy.

## Methods

### IRB approval

The study protocol “Study of aging in people with Trisomy 21: longitudinal evaluation of molecular markers and clinical-functional, neuropsychological and cognitive aspects” was approved by the Local Ethics Committee of the local health service of Bologna, Italy (1070-2021-SUPER-AUSLBO). The aim of the study is to comprehensively investigate aging in people with DS, combining the analysis of neurological, geriatric, neuropsychological and biofluids biomarkers parameters. All the participants were recruited only in the presence of a caregiver and gave their written informed consent to study participation. In case participants were unable to make decisions or presented a significant cognitive impairment, the consents were signed by a legal representative.

### Site and participant selection

Participants were recruited and evaluated at U.O.C. Clinica Neurologica Rete Metropolitana (NeuroMet), IRCCS Istituto delle Scienze Neurologiche di Bologna, with the cooperation of U.O.C. Rete Geriatrica Integrata Ospedale Territorio, AUSL Bologna, from July 2021 to December 2023. Inclusion criteria were: (1) a clinical and karyotypic diagnosis of DS, (2) to be older than 18 years old, (3) to have a written informed consent signed by the participants or by a legal representative. The only exclusion criterion applied was the presence of malignancy, in order to avoid potential confounding factors in the analysis of biomarkers of age in biofluids collected from the enrolled population.

### Clinical protocol and data collection

Neurological evaluation was carried out by expert neurologists (LS, PC) with a standardized protocol; the criteria used for the diagnosis of each clinical entity are listed in Table 1.

**Table 1:** Summary of the protocol and criteria used during neurological examination

**Neurological evaluation**	
**Medical history**	Recollection of main comorbidities Recollection of pharmacological therapy
**Neurological history**	Family history of neurological diseases Headaches Seizures or epilepsies Cerebrovascular disease Transient loss of consciousness Sleep behaviour disorders Movement disorders Psychiatric or behaviour disorders
**Cognitive status**	Ability to read and write Evaluation of the main symbolic functions (language, praxis, gnosis, memory) Deterioration of cognitive functions
**Neurologic physical exam**	Cranial nerves examination Strength examination Tone examination Sensitivity examination Reflexes examination (eye, bicipital, tricipital, patellar, plantar, grasping refelx) Coordination and equilibrium tests (finger-to-nose test, tandem walk, Romberg) Gait examination
**Diagnostic criteria**	
**Major Neurocognitive Disorder**	DSM-5
**Minor Neurocognitive Disorder**	DSM-5
**Down Syndrome Regression Disorder**	International consensus [10]
**Psychiatric Disorders**	DSM-5
**Headaches**	ICHD-3 [14]
**Epilepsy**	ILAE [15]
**Transient loss of consciousness (TLoC)**	ESC Guidelines [16]
**Sleep behaviour Disorders**	ICSD-3 [17]
**Cerebrovascular disease**	NIHSS [18]

### Extraction of data and coding

The comorbidities diagnosed during the visits, as well as other chronic diseases, were coded according to the International Classification of Diseases (ICD10), while ongoing pharmacological treatments were coded according to the Anatomical Therapeutic Chemical Classification (ATC).

Some of the elements considered during neurologic physical examination were registered into broader categories: hypokinesia, bradykinesia and rigidity were grouped as “hypokinetic disorders”; gait instability, dysmetria, cerebellar tremor were grouped as “cerebellar signs”. Tics, stereotypic movements, and nystagmus were considered separately.

Participants also underwent routinary blood tests, including complete blood cells count, electrolytes, glycaemic and lipid profile, hepatic, renal and thyroid functionality, and vitamins levels.

Clinical features were coded as categorical variables (0: absence, 1: presence), analogously to drugs (0: not treated; 1: treated) and sex (0: male; 1 female). Age, years of education, ADL and results of blood analyses were considered continuous variables.

### Statistical analysis

Binary logistic regression was used to estimate the association between clinical variables, adjusting for age. Relative odds ratio obtained from estimated effects are reported in Supplementary Table 1, together with nominal and Benjamini-Hocberg corrected *p*-values. Linear models were used to compare the levels of ADL or of biochemical measures according to the presence/absence of clinical features, correcting for age, or to compare the age of participant according to the presence/absence of clinical features. All the statistical analyses were carried out using the *glm* function in R software (version 4.2.1). A nominal *p*-value < 0.05 was considered significant.

## Results

Seventy participants (28 females, 40%) were consecutively recruited, with no patient excluded due to the presence of malignancy. Age at visit ranged from 21 to 74 years (mean age: 39.4 ± 12.0 years). The main neurological findings emerged from our evaluation are summarized in Table 2.

**Table 2:** Demographic and clinical characteristics of the cohort, including the main drugs used. *P*-values from chi-squared test (for categorical variables) and t-test (for continuous variables) are reported to compare males and females or participants younger and older than 40 years old

	Total	Males	Females		Under 40 years	Over 40 years	
	*n* = 70	*n* = 42	*n* = 28		*n* = 38	*n* = 32	
**Demographics**							
Age	39.4 ± 12.1	39.3 ± 11.6	39.5 ± 13.1	ns	30.1 ± 4.8	50.5 ± 8.1	-
Sex (female)	28 (40%)	-	-		16 (42.1%)	12 (37.5%)	ns
Education	11.3 ± 2.9	11.3 ± 3	11.5 ± 2.8	ns	13.1 ± 1.3	9 ± 2.7	< 0.01
ADL	4.7 ± 1.9	4.9 ± 1.6	4.5 ± 2.2	ns	5.5 ± 1.1	3.8 ± 2.1	< 0.01
**Main neurological comorbidities**							
Neurocognitive disorder	20 (28.6%)	9 (21.4%)	11 (39.3%)	ns	2 (5.3%)	18 (56.3%)	< 0.01
Minor	5 (7.1%)	2 (4.8%)	3 (10.7%)	ns	2 (5.3%)	3 (9.4%)	ns
Major	15 (21.4%)	7 (16.7%)	8 (28.6%)	ns	0 (0%)	15 (46.9%)	< 0.01
DSRD	5 (7.1%)	3 (7.1%)	2 (7.1%)	ns	5 (13.2%)	0 (0%)	ns
Psychiatric disorder	28 (40%)	16 (38.1%)	12 (42.9%)	ns	11 (28.9%)	17 (53.1%)	ns
Obsessive compulsive disorder	11 (15.7%)	9 (21.4%)	2 (7.1%)	ns	6 (15.8%)	5 (15.6%)	ns
Psychosis	5 (7.1%)	2 (4.8%)	3 (10.7%)	ns	2 (5.3%)	3 (9.4%)	ns
Depression	5 (7.1%)	1 (2.4%)	4 (14.3%)	ns	1 (2.6%)	4 (12.5%)	ns
Others	7 (10%)	4 (9.5%)	3 (10.7%)	ns	2 (5.3%)	5 (15.6%)	ns
Headaches	6 (8.6%)	3 (7.1%)	3 (10.7%)	ns	3 (7.9%)	3 (9.4%)	ns
Epilepsies	5 (7.1%)	1 (2.4%)	4 (14.3%)	ns	3 (7.9%)	2 (6.3%)	ns
TLoC	20 (28.6%)	10 (23.8%)	10 (35.7%)	ns	13 (34.2%)	7 (21.9%)	ns
Sleep disorders	6 (8.6%)	5 (11.9%)	1 (3.6%)	ns	3 (7.9%)	3 (9.4%)	ns
Cerebrovascular diseases	(0%)	(0%)	(0%)	ns	(0%)	(0%)	ns
**Neurological physical examination**							
No significant signs	28 (40%)	18 (42.9%)	10 (35.7%)	ns	16 (42.1%)	12 (37.5%)	ns
Hypokinetic disorder	20 (28.6%)	13 (31%)	7 (25%)	ns	6 (15.8%)	14 (43.8%)	0.02
Stereotypic movements	16 (22.9%)	11 (26.2%)	5 (17.9%)	ns	8 (21.1%)	8 (25%)	ns
Tics	8 (11.4%)	4 (9.5%)	4 (14.3%)	ns	6 (15.8%)	2 (6.3%)	ns
Tremor	2 (2.9%)	1 (2.4%)	1 (3.6%)	ns	0 (0%)	2 (6.3%)	ns
Cerebellar signs	3 (4.3%)	2 (4.8%)	1 (3.6%)	ns	2 (5.3%)	1 (3.1%)	ns
Nystagmus	10 (14.3%)	7 (16.7%)	3 (10.7%)	ns	7 (18.4%)	3 (9.4%)	ns
**Main clinical comorbidities**							
Visual impairment	40 (57.1%)	28 (66.7%)	12 (42.9%)	ns	22 (57.9%)	18 (56.3%)	ns
Hearing impairment	9 (12.9%)	7 (16.7%)	2 (7.1%)	ns	3 (7.9%)	6 (18.8%)	ns
Thyroid disease	37 (52.9%)	20 (47.6%)	17 (60.7%)	ns	18 (47.4%)	19 (59.4%)	ns
Heart disease	15 (21.4%)	9 (21.4%)	6 (21.4%)	ns	9 (23.7%)	6 (18.8%)	ns
Obesity	17 (24.3%)	7 (16.7%)	10 (35.7%)	ns	4 (10.5%)	13 (40.6%)	< 0.01
**Main drugs**							
Thyroid therapy	36 (51.4%)	20 (47.6%)	16 (57.1%)	ns	17 (44.7%)	19 (59.4%)	ns
Antidepressants	17 (24.3%)	9 (21.4%)	8 (28.6%)	ns	7 (18.4%)	10 (31.3%)	ns
Antiepileptics	5 (7.1%)	1 (2.4%)	4 (14.3%)	ns	3 (7.9%)	2 (6.3%)	ns
Antipshychotics	18 (25.7%)	12 (28.6%)	6 (21.4%)	ns	6 (15.8%)	12 (37.5%)	ns
Sedative hypnotics	5 (7.1%)	2 (4.8%)	3 (10.7%)	ns	2 (5.3%)	3 (9.4%)	ns
Mood stabilizers	4 (5.7%)	3 (7.1%)	1 (3.6%)	ns	2 (5.3%)	2 (6.3%)	ns

Forty-five participants (64%) did not present cognitive deterioration. Neurocognitive Disorder (NcD) was detected in 20 subjects (28.6%). Fifteen participants matched the criteria for Major NcD (21.4%), while 5 (7.1%) were diagnosed with Minor NcD. Five participants (7.1%), aged under 40 years, received a diagnosis of DSRD.

Psychiatric disorder was found in 28 (40%) participants. The diagnostic orientation was obsessive-compulsive disorder for 11 subjects (15.7%), psychotic disorder for 5 subjects (7.1%), and depressive disorder for 5 subjects (7.1%). Seven participants (10%) presented behavioural disorders which could not be clearly identified in specific DSM-5 diagnostic entities or in other clear clinical entities. Twelve participants (17%) with psychiatric disorders also presented cognitive decline (11 of them were also diagnosed with Major NcD and 1 with Minor NcD).

Other neurological comorbidities frequently detected were: (1) episodes of Transient Loss of Consciousness (TLoC) (*n* = 20, 28.6%), with 6 of these patients presenting cardiopathy; (2) headaches (*n* = 6, 8.6%) which, as much as we could determine from participants’ medical history, were attributed in all cases to Infrequent Episodic Tension-type Headache; and (3) epilepsies (*n* = 5, 7.1%). Two subjects presented an onset with tonic-clonic seizures after the age of 40. Six participants (8.6%) out of seven who underwent polysomnography before our evaluation were diagnosed with Obstructive Sleep Apnoea Syndrome (OSAS). Even if forms of alterations in the sleep-wake cycle and nighttime snoring were commonly observed, no other sleep disorders were diagnosed according to the ICSD-3 criteria. No one of the participant’s reported acute neurologic deficit nor cerebrovascular episodes were ever diagnosed.

Twenty-eight participants (40%) showed no remarkable sign at neurological physical examination, in 7 of these only a slightly wide-based gait was detected. The main findings during physical examination were signs of hypokinetic disorders involving 20 participants (28.6%), with no participant being diagnosed with parkinsonism. Sixteen subjects (22.9%) showed stereotypic movements of the trunk, upper and lower limbs. Eight subjects (11.4%) had tics, always localized in the oro-facial area. Ten subjects (14.3%) presented second or third grade nystagmus: 7 of them also presented visual deficits while one of them presented cerebellar signs associated. Information about the age at the onset of these disorders were obtained sporadically.

The most common drugs used by participants in our cohort were levothyroxine and psychotropic drugs (Table 2).

There were no sex differences in the frequency of any of the neurological comorbidities and findings described above (Table 2). Major NcD and hypokinetic disorders were more likely to be found in subjects older than 35 years (*p*-value < 0.01 and *p*-value = 0.02, respectively). Subsequently, we conducted a more in-depth analysis of the associations with demographic features (Supplementary Table 1). As expected, the diagnosis of Major NcD was associated with higher age (*p*-value < 0.01) and lower levels of ADLs (*p*-value < 0.01). Similar associations were found for participants with psychiatric disorder (association with age: *p*-value = 0.02; association with low ADL levels: *p*-value < 0.01). Signs of hypokinetic disorders were more likely to be found in subjects with higher age (*p*-value < 0.01), lower levels of ADLs (*p*-value = 0.03) and lower levels of education (*p*-value < 0.01). Significantly lower ADLs levels were also found in participants with DSRD (*p*-value = 0.05).

**Fig. 1:**
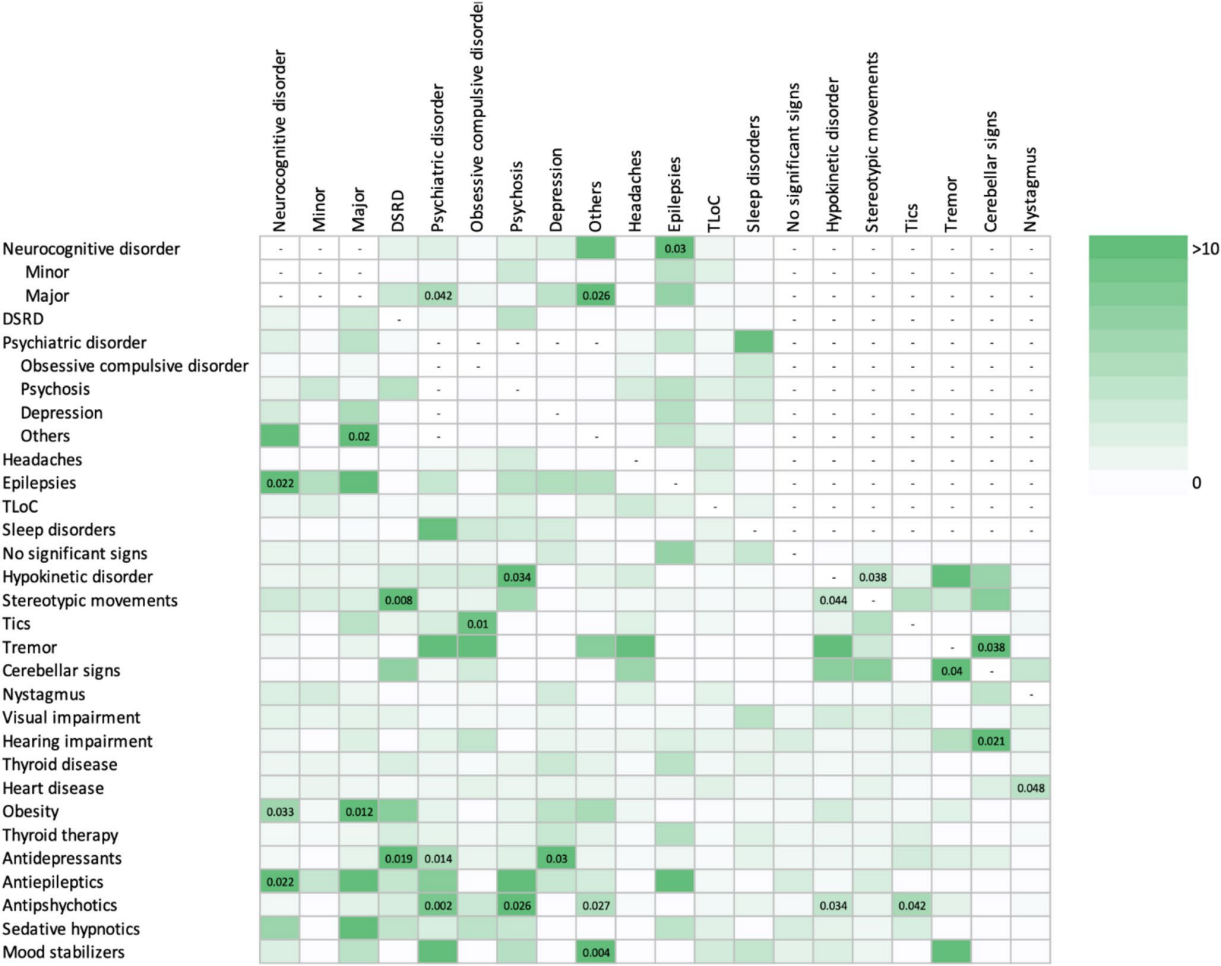
Co-occurrence of neurological phenotypes and other clinical comorbidities. The heat map graphically represents the relative odds ratio obtained from estimated effects reported in Supplementary Table 1. Significant *p*-values are reported

When considering the co-occurrence of different neurological phenotypes (Supplementary Tables 1 and Fig. 1), we found that NcD and epilepsy tend to co-occur (*p*-value = 0.02). Participants with DSRD were more prone to present stereotypic movements during our examination (*p*-value < 0.01). The diagnosis of psychiatric disorder was found to be more frequent in subjects with Major NcD (*p*-value = 0.04). Obsessive-compulsive disorder was commonly associated with tics (*p*-value = 0.001). Participants with psychotic disorder and participants in treatment with antipsychotics usually exhibited hypokinetic disorders during the examination (*p*-value = 0.03). Interestingly, stereotypic movements tend to co-occur with hypokinetic symptoms (*p*-value = 0.04). When considering the co-occurrence with other clinical comorbidities, we found that participants with NcD were more likely to be obese (*p*-value = 0.01) and cerebellar signs were associated with hearing impairment (*p*-value = 0.03).

**Fig. 2:**
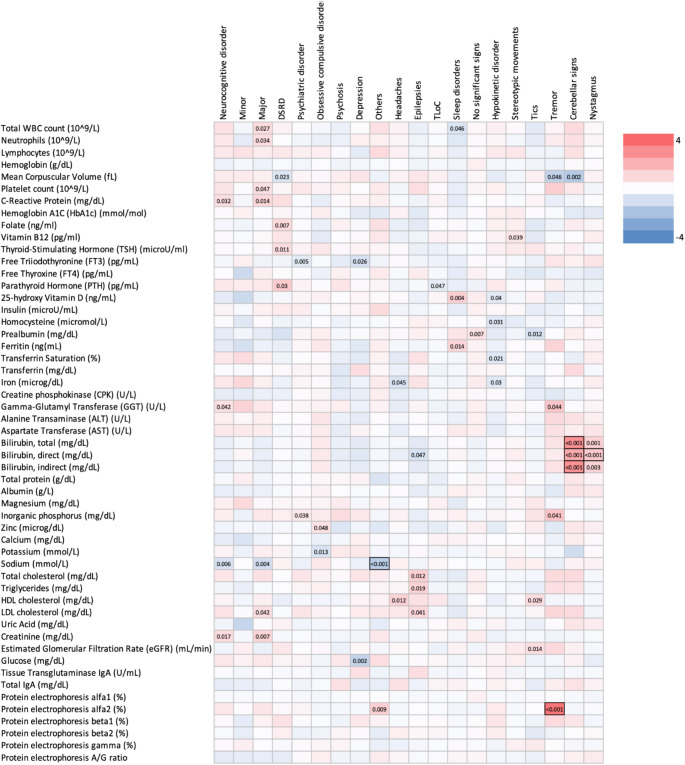
Association of neurological phenotypes with routinary blood test results. The heat map graphically represents the estimated effects reported in Supplementary Table 1. Significant *p*-values are reported, and associations having a q-value < 0.05 are highlighted

Finally, we considered the associations of the main neurological comorbidities and findings described above with routinary blood test results, which were available for 62 subjects (Supplementary Tables 1 and Fig. 2). Presence of DSRD was associated with higher levels of serum TSH (*p*-value = 0.01) and PTH (*p*-value = 0.03). Cerebellar signs and nystagmus were strongly associated with higher levels of serum bilirubin.

## Discussion

This study aims to provide a comprehensive description of the main findings detected during a neurological evaluation of a cohort of Italian adults with DS, their reciprocal relationship and their association with clinical and routinary biochemical parameters. Previous studies have focused on specific neurological phenotypes, dementia *in primis*: in this paper we aim at providing the neurologist with a complete framework for the clinical care of this population.

In our cohort NcD was found to correlate with older age, lower level of ADLs, epilepsy and obesity. The strong association we observed between cognitive decline and epilepsy is certainly of interest, considering that in almost half of the patients the onset of seizures occurred in adulthood. This finding appears to support the hypothesis that the adult-onset of seizures in DS may be an epiphenomenon of a structural brain damage, possibly sustained by Alzheimer’s disease [19]. The association with obesity may be explained by the scarce mobility of patients with NcD, in a context of loss of autonomy and social withdrawal. However, this finding is also of interest considering both the high prevalence of obesity in DS [2] and the growing evidence regarding the impact of overweight on the risk of dementia in the general population [20]. This association deserves to be confirmed and explored in future studies.

In all the patients diagnosed with DSRD the outbreak of symptoms occurred between the second and third decade of life, with a rapid decline in cognitive and functional abilities over few months. In all the subjects with DSRD it was possible to find a traumatic event at the outbreak of clinical manifestations, such as the end of high school, a relocation, or a surgical intervention. Our data also confirm the high prevalence of stereotypic movements in this group of patients [10, 21]. The diagnosis of DSRD is associated with increased serum and PTH levels. A reduction in vitamin D levels in the same patients has been described in the literature [22], suggesting the existence of a subclinical alteration in the same metabolic pathway. The same paper reports thyroid dysfunctions in these patients: we also found an association with abnormal blood TSH levels. Further studies are needed to confirm and extend these data as well as to understand the pathogenetic mechanisms underlying this disorder.

Identifying psychiatric disorders in people with ID can be demanding [4, 11, 23]. Based on our experience, the limited applicability of DSM-5 criteria in this population led us to categorize patients into macro-groups of psychiatric disorders, without being able to make more precise diagnoses. Obsessive-compulsive disorder was the most frequent. The main characteristics detected were repetitiveness of bizarre behaviours and limited mental flexibility. Depressive disorder in our cohort occurred with typical symptoms, such as apathy, abulia and withdrawal. Participants diagnosed with psychotic disorder exhibited clear hallucinations, delusions, and soliloquies. Given the clinical complexity of DS during adulthood and the difficulty in distinguishing various comorbidities impacting the behavioural framework, a reasonable approach we follow in our clinical practice is to diagnose and treat psychiatric disorders when the distinctive features of each of them significantly affected the patient’s quality of life.

Our work highlights that the onset of psychiatric disorders is closely related to the patients age and that patients with Major NcD, although with a weak correlation, are more prone to present psychiatric disorders. Interestingly, a stronger association was found between Major NcD and psychiatric disorders classified as “others”. As discussed above, these behavioural disorders were not clearly structured or attributable to a specific clinical entity proposed in the DSM-5, and we can hypothesize that they are an epiphenomenon of cognitive deterioration. To date, little is known about the relationship between psychiatric disorders and cognitive decline, both in DS and in the general population, although it is likely that it results from a complex interaction between anatomical, biological and psychosocial factors. This area that deserves future investigation. At the same time, the lack of association between the other types of psychiatric disorders and NcD suggests that the behavioural disorders documented in clinical practice in some patients may be considered as independent clinical entities. Psychiatric disorders in DS are relatively unexplored, although the presence of behavioural disorders is well documented [23]. This is primarily due to the lack of adequate and widely accepted diagnostic tools. Furthermore, the clinical overshadow of ID and cognitive decline, make the recognition of such disorders notably challenging [24].

TLoC were found to be very common and, interestingly, did not correlate with any of the variables considered suggesting, together with their high prevalence found in our cohort, that these episodes may be the expression of a constitutive condition. In recent studies, DS was found to be associated with impaired blood pressure and heart rate response to sympathoexcitation [25] and with reduced work capacity [26], suggesting the existence of an underlying autonomic dysfunction. Also, hypotension is a nearly constant condition in individuals with DS: although it is mostly reported in the paediatric age [27], it was also frequently found during our evaluation. Finally, only few of the patients with TLoC presented cardiopathy.

The prevalence of epilepsy found in our cohort is slightly lower compared to that described in the literature [28]. Participants with epilepsy presented higher serum levels of triglycerides, LDL and total cholesterol compared to the rest of the cohort, reasonably due to effects of antiepileptic drugs [29].

Based on our experience, we believe that the prevalence of OSAS is largely underestimated in our cohort [2] mainly due to the small number of participants undergoing polysomnography despite the high prevalence of nighttime snoring. The negative effects of OSAS on cognition is well known. Although this influence is mainly documented in children and young adults with DS [30], a prompt diagnosis and treatment of this comorbidity in older patients is recommended.

None of the subjects enrolled had ever been diagnosed with a stroke or cerebrovascular disease, nor participants or caregivers reported the occurrence of signs or symptoms referable to cerebrovascular events. Despite the prevalence of cardiovascular risk factors in DS seems to be comparable to that in general population, some papers highlighted a higher risk of major cerebrovascular events, essentially due to cardioembolic events in association with congenital heart disease [31, 32]. The absence of cerebrovascular event in our cohort is reasonably due to the limited size of our sample.

The unexpectedly high prevalence of movement disorders is a notable observation, especially given that studies focusing on DS neurological pathology seldom described such findings. This can be explained by the complexity of the syndromic condition which can focus the attention of physicians and caregivers on more impactful comorbidities. To the best of our knowledge, two papers systematically assessed movement disorders in DS, describing the association between movement disorders, in particular dyskinesias, and cognitive impairment severity [12, 13]. Hypokinetic disorders were described by Haw with a prevalence comparable to our cohort (33% of the patients presented bradykinesia and 4% clear parkinsonism), although in none of our participants hypokinesia or bradykinesia were associated with rigidity. In most cases, it was not possible to define the age of onset of movement disorders, but we found that hypokinetic symptoms were more frequent in older individuals. Structural brain alterations, such as grey matter atrophy, white matter degeneration, basal ganglia calcifications, are a common finding in people with DS and are often interpreted as signs of brain aging. However, the relationship between these abnormalities and neurological phenotype has yet to be fully clarified [33–35]. Moreover, it should be noted that hypokinetic symptoms were also associated with the diagnosis of psychotic disorders (which tended to be greater in the older group) and, consequently, with the assumption of antipsychotic drugs, which can justify such findings. Stereotypic movements are defined as purposeless, repetitive, coordinated and bizarre movements. In our cohort the prevalence of this finding was lower than what reported by Haw. Notably, stereotypies were detected in all the patients with DSRD: previous papers indeed consider this disorder as a major feature of DSRD [21].

Tics are sudden, brief movements or sounds involving discrete muscle groups. Despite their prevalence is significantly lower than what was found by other authors [12, 13], reasonably due to the differences in assessment methods, our findings seem to confirm that subjects with DS are particularly prone to develop involuntary movements in the oro-facial area. Notably, tics were strongly associated with obsessive-compulsive behaviours. This association is, indeed, described in the general population [36]. Nystagmus seems to be a frequent finding in DS, despite being almost exclusively described in children [37, 38]. In our cohort, nystagmus tended to occur frequently in patients with cardiopathy: previous studies found contrasting results regarding this association [39, 40]. Nystagmus and cerebellar signs were strongly associated with higher serum bilirubin levels. Most of these patients presented slighted increased levels of indirect and total serum bilirubin, without any strong association with drug assumption and hepatic damage (data not shown). These data suggest the existence of a constitutive anomaly of bilirubin metabolism. Bilirubin is known to have neurotoxic effects especially during brain development, including damage to the cerebellum [41], and neonatal jaundice is more common in infants with DS [42]. Even if some authors suggest that nystagmus may origin form cerebellar dysfunctions in children with DS [43], there is still lack of information about its occurrence during adulthood and the scarce association between nystagmus and cerebellar signs in our cohort led us to hypothesize the existence of different pathogenetic mechanisms.

Lastly, it is worth mentioning the common use of psychotropic drugs in our cohort, further complicating the global neurological assessment and underlying the need for specialized care.

## Conclusions

In conclusion, in this paper we provide a systematic description of the main neurological findings in a cohort of adults with DS. We acknowledge that our study has some limitations. The monocentric and retrospective design of the study, along with the limited size of our sample, may limit the generalizability of our findings. The lack of specific diagnostic criteria for psychiatric disorders in this population exposes the diagnosis to a certain degree of subjectivity, potentially affecting the reproducibility of the results. The lack of standardized criteria also exposes the study to a severity bias. Finally, no instrumental characterizations are available for the subjects included in the study.

Notwithstanding, our findings confirm and further enrich current knowledge on neurological phenotypes of adults with DS. Neurocognitive and psychiatric disorders were common comorbidities, typically in individuals older than 40 years of age. Transient loss of consciousness was frequently reported in participants’ anamnesis, suggesting the need for a deep comprehension of autonomic functionality in DS. Signs of movement disorders were a common finding during physical examination, particularly hypokinetic disorders, stereotypic movements, and nystagmus. Co-occurrence analysis found several associations that may better address future research in understanding the pathogenesis of such findings.

In summary, the high prevalence of neurological comorbidities and symptoms highlights the need for a specialized care for people with DS during adulthood.

## Electronic Supplementary Material

Below is the link to the electronic supplementary material.

Supplementary Material 1
